# Ensemble Distribution Modeling of the Globally Invasive Asian Cycad Scale, *Aulacaspis yasumatsui* Takagi, 1977 (Hemiptera: Diaspididae)

**DOI:** 10.3390/insects16101016

**Published:** 2025-09-30

**Authors:** Samuel Valdés-Díaz, Reyna Tuñón, Dilma Castillo, Alieth Sanchez, Brenda Virola-Vasquez, Patricia Esther Corro, Francisco Serrano-Peraza, Bruno Zachrisson, Jose Loaiza, Rodrigo Chang, Luis Fernando Chaves

**Affiliations:** 1 Programa Centroamericano de Maestría en Entomología (PCMENT), Universidad de Panamá, Ciudad de Panamá Estafeta Universitaria, Apartado 3366, Panamá 4, Panama; reyna070127@gmail.com (R.T.); dilmac-09@hotmail.com (D.C.); sanchezalieth920@gmail.com (A.S.); bsvirola@gmail.com (B.V.-V.); ephysteris04@gmail.com (P.E.C.); fcoserrano90@gmail.com (F.S.-P.); bazsalam@gmail.com (B.Z.); jloaiza@indicasat.org.pa (J.L.); rodrigoachang@gmail.com (R.C.); lfchavs@gmail.com (L.F.C.); 2 Biodiversity Consultant Group (BCG), Hato Pintado, Ciudad de Panama PA 07126, Panama; 3 Instituto de Innovación Agropecuaria de Panamá (IDIAP), Chepo, Ciudad de Panama PA 07052, Panama; 4 Instituto de Investigaciones Científicas y Servicios de Alta Tecnología, Panama, PO Box 0843-01103, Panama; 5 Department of Environmental and Occupational Health, School of Public Health, Bloomington, IN 47408, USA; 6 Department of Geography, Indiana University, Bloomington, IN 47405, USA

**Keywords:** Asian Cycad Scale, invasive species, occurrence records, habitat suitability, niche prediction, ensemble species distribution models

## Abstract

Species distribution models (SDMs) have become an important tool to inform conservation and pest surveillance programs about the potential biological invasion of insect pests. Nonetheless, to be operational, SDMs need to incorporate multiple environmental covariates and a representative number of occurrence points depicting the species’ ecological niche. The algorithm of choice, model of choice, and comparison can also have a great effect on the final prediction output. We created a dataset based on previously published records, plus 36 new occurrences and 37 environmental predictors, to generate the first global ensemble distribution model for *Aulacaspis yasumatsui*. We employed a strategy that aggregates SDMs with the best performance (i.e., greater accuracy) from six different algorithms, resulting in an averaged and weighted model, i.e., the ensemble model. We then selected models from algorithms whose true skill statistic (TSS) was above 0.5 in order to map the potential global distribution of *A. yasumatsui*. Our results suggest that covariate selection and the individual model algorithms used in the ensemble may be more important for achieving an accurate SDM than the number of occurrence points.

## Simple Summary

*Aulacaspis yasumatsui* is an invasive scale insect of great economic importance to the horticulture industry, affecting plant species in the *Cycas* genus. Prior attempts to model the ongoing spatial expansion of *A. yasumatsui* were based on the MaxEnt algorithm and WorldClim bioclimatic covariates to predict shifts in its distribution owing to future climate scenarios. Here, we develop the first ensemble species distribution model including recent occurrence records and environmental predictors not analyzed in previous efforts. We combine the outcomes of six different algorithms in order to improve model accuracy and minimize model error. The findings support the likely invasion of *A. yasumatsui* in tropical and subtropical areas of Africa and Central America. Dispersal is also highly possible through northern areas of Europe and China.

## 1. Introduction

Invasive scales, aphids, whiteflies, and leafhoppers have become significant global threats to agriculture and horticulture, driven largely by the movement of exotic plants and agricultural products through increased international trade [1]. Biological traits of these invasive insects, including high reproductive capacity, broad host ranges, and climatic adaptability, enable their rapid establishment and spread into novel environments, necessitating enhanced monitoring and predictive modeling to mitigate their ecological and economic impacts [2].

*Aulacaspis yasumatsui* Takagi, 1977 (Hemiptera: Diaspididae), commonly known as the Asian Cycad Scale, is an armored scale insect with a particular preference for *Cycas revoluta* Thurnberg, 1782, a member of the family Cycadaceae that has been hypothesized as its main host plant [3]. The first observation of *A. yasumatsui* feeding on *Cycas revoluta* was at the type locality of Bangkok, Thailand, in 1972 by Takagi [4]. According to the original description, *A. yasumatsui* is native to the tropical and subtropical areas of Southeast Asia. However, the recent accumulation of new records from around the world has confirmed that *A. yasumatsui* is also able to feed on plants of the family Zamiaceae, affecting mainly the genera *Dioon*, *Encerphalartos*, *Macrozamia*, *Microcycas*, and *Stangeria* [5,6,7,8].

*Aulacaspis yasumatsui* has been classified as an exotic invasive pest according to the Crop Protection Compendium of CABI, 2021. *Aulacaspis yasumatsui* has been reported in several countries where *Cycas revoluta* was previously introduced through the cycad horticulture trade [8]. A recent study on the genus *Aulacaspis*, including *yasumatsui*, highlights the economic importance of this insect group as pests of sugarcane, ornamental grasses, and herbs on a global scale. Due to the increased international trade of *Cycas revoluta*, the expanded distribution of *A. yasumatsui* now includes records from across the Americas [9,10,11,12,13,14,15,16], Asia [17,18,19], Europe [19,20,21], and Africa [22,23,24]. The new distribution records warn non-native countries about the negative impact that *A. yasumatsui* could have on the Neotropical members of the family Zamiaceae, of which many species have been declared as critically endangered or vulnerable by the IUCN Red List of Threatened Species, and call for special considerations in the elaboration of contingency plans against the invasion of this insect pests. Despite the increasing number of countries being invaded by *A. yasumatsui* in the last 10 years and the hidden economic hazard posed by this pest to the cycad horticulture industry around the world, little effort has been made towards gaining a better understanding of the potential spread of this species outside its native range [8,18]. In particular, we know little about which ecological variables, measured with remote sensing, other than climatic factors might impact the likely geographic expansion and habitat suitability of this species globally [25].

Species distribution models (SDMs) are widely used in ecology to predict the distribution of suitable habitat for a particular species of interest [26,27]. Traditionally, species distribution models (SDMs) have relied upon species occurrence points and values of selected environmental covariates as data inputs, which has two implications for their ability to estimate habitat suitability parameters [28]. Species occurrence datasets may fail to adequately capture the fundamental niche for taxa with a high dispersal capability, which can lead to populations in sink habitats [29], whereas dispersal limitation may exclude species from large areas of suitable habitat [16,30]. These issues limit the ability to fully estimate fundamental niches from observed species occurrence types of data. A way to deal with these limitations is to evaluate and compare the outcomes of several model algorithms and select the best prediction based on a set of performance metrics [31,32,33]. Alternatively, the selection of just one single ensemble model combining the strengths and caveats of all models can be used to make accurate estimates of species distribution, provided that the model is stable and robust.

Previous efforts to predict the continental distribution of *A. yasumatsui* through SDMs relied on the MaxEnt algorithm, a machine learning method that predicts a species’ existence in a coordinate-based space built with the species occurrence (or presence-only) points and the environmental background where the species has not been reported [34,35]. This approach makes it almost impossible to assess specificity since no information on species absence is used [28,36,37]. Ensemble models of species niche distributions are commonly used to mitigate the impact of model performance variability across multiple species, localities, and datasets because no single method consistently outperforms those available, and ensemble methodologies can increase the accuracy of SDMs and reduce their error [38,39].

The performance of species distribution models (SDMs) is influenced not only by the quantity of occurrence records but also critically by their spatial arrangement and the design of the model evaluation [30]. Traditional evaluations have demonstrated that model accuracy can be artificially inflated due to the spatial clustering of data points and the methods used for validation, with limited gains achieved by simply increasing the sample size when data are dense yet spatially biased [26,34]. Presence-only modeling approaches, in particular, require careful handling of absence or pseudoabsence data to prevent overly confident predictions, while recent studies highlight that failing to explicitly address sampling bias and evaluation strategies can lead to unrealistic SDMs [30,34].

Equally vital to SDM accuracy is the selection of predictor variables and the modeling approach. Ecological theory and empirical benchmarks underscore the importance of choosing predictors that are both ecologically meaningful and minimally redundant, as well as managing collinearity among variables, to enhance the model’s ability to generalize across different spatial and temporal contexts [22,23,37]. The choice of algorithm also plays a significant role, affecting the flexibility of response shapes and the risk of overfitting. Ensemble modeling frameworks that integrate diverse algorithms, such as Generalized Linear Models, Boosted Regression Trees, Random Forests, and Artificial Neural Networks, can improve prediction stability when combined with rigorous model selection criteria and robust evaluation metrics like the Area Under the Curve (AUC) and true skill statistic (TSS) [29,31,37]. Building upon these foundations, this study explicitly investigates how the number of occurrence records, reduction in covariates, and use of an ensemble of several algorithms influence the predictive performance and geographic realism of an SDM for the invasive scale insect *Aulacaspis yasumatsui.*

Here, we created a dataset based on 158 *A. yasumatsui* occurrence records, including 122 previously reported and analyzed occurrences, plus 36 new occurrences. As covariates, we included 21 WorldClim variables plus 18 additional environmental predictors, including remotely sensed data not considered in previous *A. yasumatsui* SDM efforts. We started with 37 covariates and 10 sets of pseudoabsence points, i.e., simulated locations where the species is presumed absent across the planet, where the number of pseudoabsences was the same as that of *A. yasumatsui* occurrences. We went through a process of model selection in order to reduce the number of covariates, thus avoiding the overfitting of the SDM. For the ensemble model, we employed a strategy that aggregates niche models with the best performance (i.e., greater accuracy) from six independent algorithms, resulting in an averaged and weighted model based on each model’s predictive performance, which has been shown to improve ensemble predictions [39,40]. We repeated this procedure for a model based on the same set of occurrences previously analyzed [32], which we compared with our larger dataset and whose analysis showed that model selection might be more important than the abundance of occurrence points to model the global distribution of an insect.

Here, our goal was to generate the first robust and comprehensive global ensemble distribution model for *A. yasumatsui* that can be used as a resource for monitoring, preventing, and controlling the threats imposed by this insect pest to native host plants and the horticultural industry around the world, specifically *Cyca* spp. and Zamiaceae.

## 2. Materials and Methods

### 2.1. Occurrence Records

We compiled *Aulacaspis yasumatsui* records from GBIF (GBIF Occurrence Download. Available online: https://doi.org/10.15468/39omei accessed via GBIF.org on 5 March 2022), CABI, and the peer-reviewed literature (1977–2022); taxonomy was harmonized to *A. yasumatsui* Takagi; ambiguous “cycad scale” mentions were excluded unless explicitly confirmed. Coordinates were standardized to WGS84 and filtered to remove zeros, country/province or institution centroids, marine points, and entries discordant with verbatim locality. We kept only records with ≤5 km coordinate uncertainty or demonstrably precise localities; exact duplicates were removed using the “spThin” function from the R package spThin (version 0.2.0) [41], and multiple points per 2.5′ grid cell were collapsed to a single occurrence. To limit clustering at the environmental resolution (~4.6 km at the equator), we applied 5 km spatial thinning. 

### 2.2. Data Sources and Processing of Environmental Data

We built a multilayer raster incorporating 37 environmental covariates to fit species distribution models for *A. yasumatsui* under current environmental conditions. These predictors were chosen due to their general physiological significance to insects and because recent research has revealed that such covariates could be important in shaping insect spatial distributions [42].

Environmental predictors were obtained from WorldClim v2.1 [43] at a 2.5′ resolution (19 bioclimatic variables derived from 1970 to 2000 monthly temperature and precipitation) and from Google Earth Engine (GEE [44,45]). From GEE, we accessed TerraClimate (monthly climate and climatic water balance at 1/24° ≈ 4 km; 1958–31 December 2021 [46]), the NOAA Climate Data Record (CDR) of AVHRR Normalized Difference Vegetation Index (NDVI) based on daily images from 24 June 1981 to 31 December 2021 at a resolution of 0.05° ≈ 5.66 km [47], MODIS/Terra Land Surface Temperature/Emissivity (daily, 1 km resolution, 1st January 2002 to 31 December 2021 [48]), GTOPO30 (completed in 1996, global elevation at 30-arc-second ≈ 1 km [49]), and ALOS PALSAR Forest/Non-Forest (25 m, annual estimates from 2009 to 2017 [50]). To represent forest cover, we used PALSAR’s categorical layer (forest, water, non-forest) which follows the FAO criterion (>0.5 ha with >10% canopy cover). The time series of rasters collected with the Google Earth Engine were further processed with the reducer command to estimate the median for each pixel. All rasters were re-projected to WGS84 (EPSG:4326), snapped to a common 2.5′ grid (~4.6–5 km at the equator), and resampled using bilinear interpolation for continuous variables (WorldClim, TerraClimate, NDVI, elevation) and nearest neighbors for categorical data (PALSAR) [51,52]. Higher-resolution layers were aggregated to 2.5′ by area-weighted mean (continuous) or majority class (categorical). 

### 2.3. Ensemble Algorithms, Pseudoabsences, and Covariate Selection

To build the ensemble distribution model [53], we followed a methodology similar to the one described by Rhodes et al. [54]. We employed six algorithms for modeling the global distribution of *A. yasumatsui*. These included Logistic Generalized Linear Models (L-GLMs), Multiple Adaptive Regression Splines (MARSs), and three tree-based methods which include Classification and Regression Trees (CATs), Generalized Boosted Regression Models (GBMs), and Random Forests (RFs), and we also employed Artificial Neural Networks (ANNs). For detailed explanations of each one of these algorithms, we refer the interested reader to Rhodes et al. [54]. All analyses were completed using the biomod2 package for R [55]. This package was selected for its ability to incorporate several SDM modeling techniques and reproducibility settings. Results from all algorithms were cross-validated using fivefold cross-validation, i.e., where one fifth of the observations are left out of model fitting and used to evaluate the model each time the model is fitted using an incomplete dataset.

To fit the model, we generated 112 pseudoabsence points. While it is possible to fit SDMs with presence-only data, presence–absence data has shown superior performance [56]. However, true absence points are rare and particularly difficult to confirm for mobile species [57]. Without true absence points, we rely on an artificial set of absence points, termed “pseudoabsence points” [57,58]. There are many different strategies for generating these points, so we refer to the suggestions in [57], which details the best sampling method based on the algorithms used. We started with 37 covariates and went through a process of model selection in order to have a reduced number of covariates that avoided overfitting. For the first stage of covariate selection, we generated 10 sets of 112 random pseudoabsence points, a number equal to the occurrences we had after thinning the dataset and a balanced number (i.e., number of occurrences = number of pseudoabsences) that ensures the best predictive performance for machine learning algorithms [59,60,61]. We then ran each one of the six algorithms three times, and each of the three times, we included 100 permutations for each covariate at a time. These permutations allowed us to estimate covariate importance, a measurement of the drop in explained variance or prediction accuracy by a given covariate, which does not involve the use of Pearson’s correlation [57,62].

Based on this preliminary analysis, we chose to include all covariates whose importance was above 5%. We repeated this process one extra time, until we selected seven covariates (Table 1). For the seven selected covariates, we generated ten additional pseudoabsence datasets of 112 locations, this time employing the Surface Range Envelope (SRE) method in the biomod2 package. The SRE method was used to identify a range of suitable environmental conditions [63]. Pseudoabsence points were then sampled randomly outside of that area, as they were considered environmentally dissimilar to the location of presence points. The resulting datasets of absences and SRE-generated pseudoabsences were run one time for each one of the six algorithms, including 100 permutations for each covariate, to assess their importance.

To visualize the results from the median of the 10 datasets used to generate the ensemble, we employed strip plots. These plots show curves for the probability of occurrence, as a function of each covariate, for each algorithm used to build the ensemble SDMs. The strip plots were generated by producing a prediction from a model using a new dataset in which only one covariate was allowed to vary in a sequence between the minimum and maximum extremes, while the other covariates were fixed at their median values [64].

To evaluate the performance of ensemble SDMs and covariate selection, we employed the Area Under the Curve (AUC) and the true skill statistic (TSS) evaluation statistics. The AUC estimates an algorithm’s ability to correctly differentiate between presence and absence locations, with a value of 0.5 suggesting that model performance is no better than random chance. Meanwhile, the TSS works similarly and is equal to the sum of model sensitivity and specificity minus one [55]. Similar, or higher, AUC and TSS values suggest that models have similar, or improved, performance. We used models from algorithms whose TSS was above 0.5 to generate global ensemble SDM maps of *A. yasumatsui* based on the 112 occurrence records we compiled. As all individual models had a TSS > 0.5, we weighted all the resulting 60 projected predictions for *A. yasumatsui* habitat suitability, using the TSS value from each individual model as a proportional weight in the ensemble. We employed the TSS as this metric is independent of the prevalence of occurrences while being interpretable like the AUC [65]. Finally, for comparison, we also fitted the six algorithms ten times, employing the seven selected covariates, to a restricted dataset that only considered the 86 occurrence records from [35] and 86 pseudoabsences also generated using the SRE method.

## 3. Results

**Figure 1:**
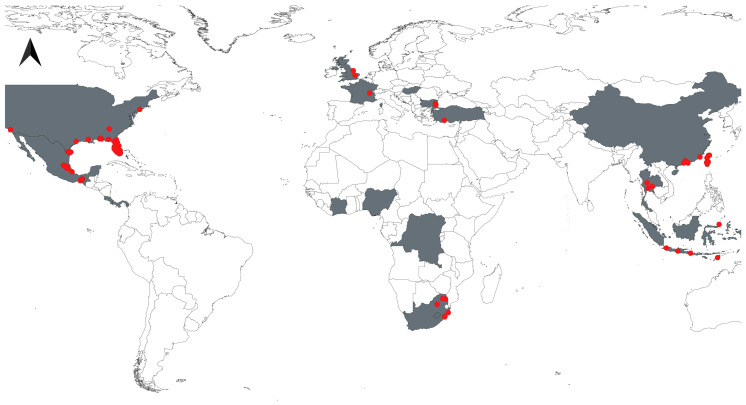
Map showing the geographic locations of the invasive Asian Cycad Scale *Aulacaspis yasumatsui,* red dots indicate occurrence points after data cleaning and thinning. The final data coverage includes 20 countries with 112 points.

**Figure 2:**
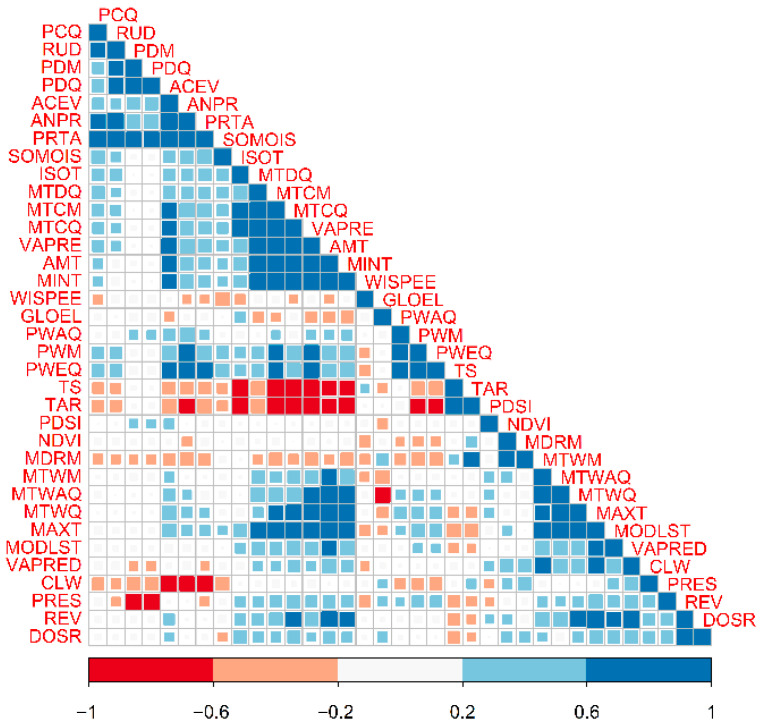
Pairwise Pearson’s correlation between environmental covariates at the global occurrence points for the invasive Asian Cycad Scale *Aulacaspis yasumatsui*. Correlations have been clustered to ease the visualization of groups of highly correlated covariates. High correlations represent r values −0.6 > r > 0.6.

We obtained 122 occurrence points for *Aulacaspis yasumatsui*, including 36 new records not included in the 86 records from a prior global analysis [35]. Following data cleaning and thinning, 112 records from 20 countries remained, the majority of which were distributed in the United States (46 occurrences), Mexico (15 occurrences), and Taiwan (10 occurrences), see Figure 1. The correlations between the various covariates at the pixels where *A. yasumatsui* was collected are presented in Figure 2.

**Figure 3:**
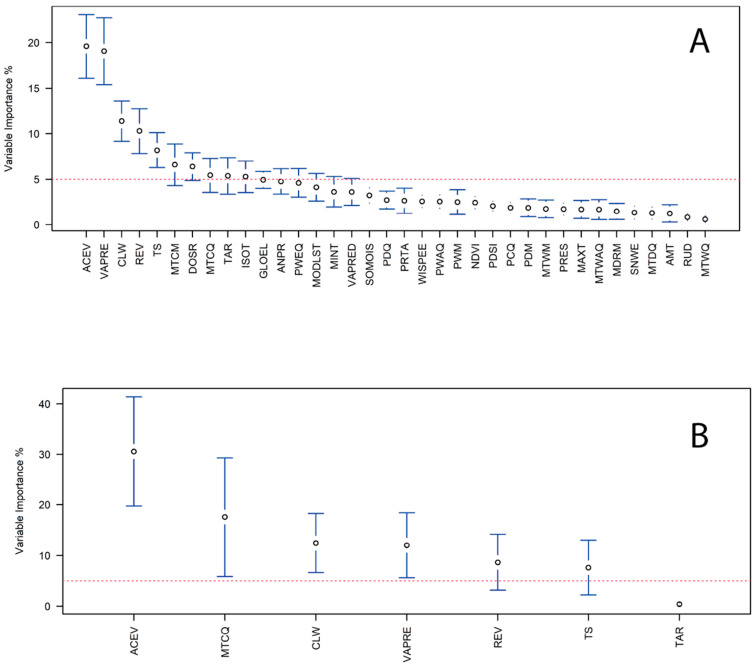
Covariate importance in the ensemble species distribution model of the invasive Asian Cycad Scale *Aulacaspis yasumatsui*: (**A**) All covariates used for initial model selection; the red dashed line indicates the 5% importance threshold. (**B**) Covariates selected for the final ensemble model. Red dashed line indicates 5% variable importance threshold.

The Pearson’s correlogram (Figure 2) shows clusters of high covariate correlations (r > 0.6), particularly for the temperature-related covariates, i.e., the Annual Mean Temperature (AMT), Mean Temperature of the Wettest Quarter (MTWQ), Mean Temperature of the Coldest Quarter (MTCQ), Minimum Temperature of the Coldest Month (MTCM), and Minimum Temperature (MINT). These patterns of association called for a process of covariate selection based on covariate importance, whose results are presented in Figure 3.

Of the thirty-seven environmental covariates initially investigated, only seven were selected for inclusion in the final ensemble species distribution model. During model selection, covariate importance was over 5% for only ten environmental covariates (Figure 3A). After model selection (Figure 3B), the Actual Evapotranspiration (ACEV) and Mean Temperature of the Coldest Quarter (MTCQ) were the two most important covariates with roughly 30% and 18% of importance, respectively. Of the five remaining, the next most important environmental covariates were the Climate Water Deficit (CLW), Vapor Pressure (VAPRE), Reference Evapotranspiration (REV), Temperature Seasonality (TS), and Temperature Annual Range (TAR) (Figure 3B).

Species distribution models were created using six different algorithms and 112 occurrences to estimate habitat suitability, including 36 new records of *A. yasumatsui*. The ROC and TSS scores for the models reported in Table 2 indicate better performance for the final model with seven covariates (Figure 3B). Random Forest (RF) and Generalized Boosted Regression Models (GBMs) showed the best performance with AUC scores of 0.99 ± 0.13 and 0.98 ± 0.01 plus TSS scores of 0.91 ± 0.08 and 0.89 ± 0.08, respectively. Artificial Neural Networks (ANNs; AUC = 0.93 ± 0.05, TSS = 0.82 ± 0.12) and Classification and Regression Trees (CTAs; AUC = 0.91 ± 0.04, TSS = 0.81 ± 0.08) also showed good performance but were, comparatively, the worst-performing algorithms. Using the same six algorithms and seven covariates but only the 86 occurrence records from Wei et al. [35] generally produced lower AUC values but higher TSS scores in all models (Table 3).

**Figure 4:**
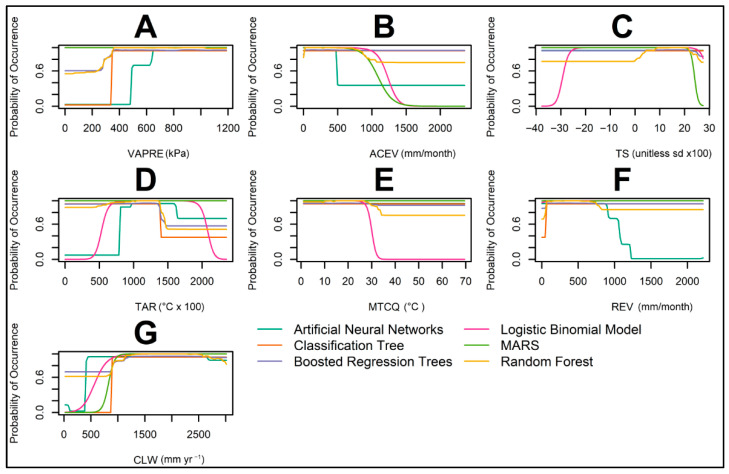
Strip plots for the predicted probability of occurrence for the invasive Asian Cycad Scale *Aulacaspis yasumatsui* using 112 occurrence records (Supplementary Material Figure S1 shows the results based on the 86 records from a previous study [35]). Media of replicated runs for each model are represented by lines. Model algorithms are color-coded. Environmental covariates included (**A**) Vapor Pressure (VAPRE), (**B**) Actual Evapotranspiration (ACEV), (**C**) Temperature Seasonality (TS), (**D**) Temperature Annual Range (TAR), (**E**) Mean Temperature of the Coldest Quarter (MTCQ), (**F**) Reference Evapotranspiration (REV), and (**G**) Climate Water Deficit (CLW).

All six modeling algorithms consistently predict an increase in the global probability of occurrence for *Aulacaspis yasumatsui* with rising Vapor Pressure (VAPRE) levels, particularly at values exceeding 250 KPa (Figure 4A). For the Logistic Binomial Model (LBM), Multivariate Adaptive Regression Splines (MARSs), and Random Forest (RF), Actual Evapotranspiration (ACEV) (Figure 4B) exhibits a strong negative relationship with occurrence probability, especially when ACEV surpasses 900 mm/month. This negative correlation is less pronounced when using Random Forest and Boosted Regression Trees (BRTs), indicating reduced model sensitivity to this covariate.

Temperature Seasonality (TS), which quantifies the extent of temperature fluctuation over a year (Figure 4C), generally demonstrates limited influence on predicted suitability across most algorithms. However, the LBM strongly favors low-TS environments, suggesting that stable annual temperature regimes may be more suitable.

The Temperature Annual Range (TAR) (Figure 4D), the differential between the warmest and coldest months, shows minimal effect on occurrence probability across the majority of models. The Logistic Binomial Model (LBM) reveals a distinct unimodal response, with habitat suitability increasing at TAR values above 400 (equivalent to 4 °C), peaking around 1000 (10 °C), and declining at values beyond 1500 (15 °C). This pattern suggests that *A. yasumatsui* may favor environments with moderate annual temperature fluctuations, while extreme variability is less suitable.

Artificial Neural Networks (ANNs) follow a similar pattern, with occurrence probability sharply rising between 600 and 1000 (6–10 °C) and then decreasing at higher TAR values. Meanwhile, MARSs and Boosted Regression Trees (BRTs) predict relatively stable suitability across the TAR spectrum, indicating a limited role of this covariate in those models. Random Forest shows high occurrence probabilities throughout most of the TAR range but exhibits a gradual decline past 1500 (15 °C), suggesting some sensitivity to higher temperature variability.

The Mean Temperature of the Coldest Quarter (MTCQ, in °C × 100) emerged as an influential predictor of *Aulacaspis yasumatsui* occurrence in select models (Figure 4E). The Logistic Binomial Model (LBM) exhibited a sharp threshold response, with suitability declining steeply above 350 (3.5 °C), suggesting a lower thermal tolerance limit during colder seasons. In contrast, models such as ANNs, MARSs, BRTs, and Classification Trees maintained high suitability across the full MTCQ range, indicating limited sensitivity to cold-season temperatures. Random Forest showed a gradual decline in suitability above 300 (3 °C).

Climate Water Deficit (CLW), a measure of atmospheric water demand unmet by available moisture, showed varying degrees of influence on the predicted occurrence of *Aulacaspis yasumatsui* across models (Figure 4G). The LBM and MARSs predicted a strong, monotonic increase in suitability with rising CLW, plateauing above 700 mm, indicating potential adaptation to drier conditions. ANNs showed a similar trend, with a sharp rise in occurrence probability at intermediate CLW values. Classification Tree models exhibited a threshold-based response, while BRTs and Random Forest maintained high suitability across the entire CLW gradient. These patterns suggest that *A. yasumatsui* may be tolerant of water-limited environments, particularly as inferred from models sensitive to continuous climatic gradients.

**Figure 5:**
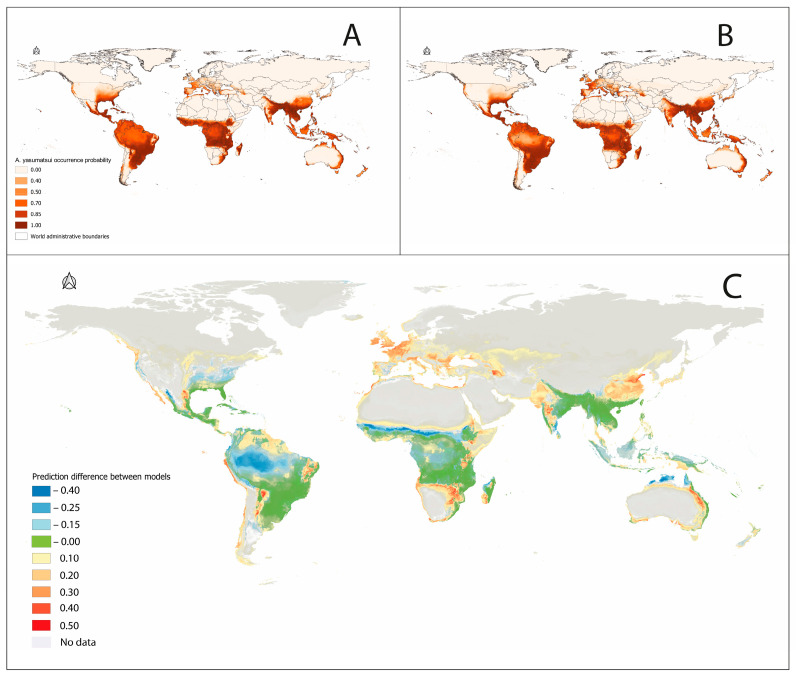
Global ensemble distribution model for the invasive Asian Cycad Scale *Aulacaspis yasumatsui*. Color indicates habitat suitability measured as probability from 0 to 1, as presented in the legend. (**A**) SDM generated with previously published data [35]; (**B**) SDM generated with 122 records of this study; (**C**) differences in predicted distribution models. Blue indicates areas with medium and high occurrence probability predicted in (**A**) but low probabilities predicted in (**B**). Red indicates areas with medium and high probabilities of occurrence in (**B**) but with low probability in (**A**).

Two final ensemble models were developed: one utilizing previously published occurrence data [25] and the other incorporating an updated occurrence dataset. These ensemble predictions are presented in Figure 5A,B, with spatial differences between models highlighted in Figure 5C. Compared to earlier modeling efforts, our updated ensemble predicts higher occurrence probabilities (depicted in warm tones) in northern Europe, the Mediterranean basin, northern China, Japan, and parts of eastern Australia. In the Americas, increased probabilities are projected along the Pacific coast from Peru to Chile, as well as across fragmented regions of the Caatinga, Chaco, and Savanna biomes and within the Cascade Range of western North America.

Conversely, our updated ensemble model predicts reduced occurrence probabilities (cool tones) relative to previous models in regions such as the Amazon basin, the Appalachian Mountains, the dry tropical zones of Africa, and northern Australia, including Arnhem Land and the York Peninsula. Regions with little to no difference between models are represented in green.

Despite both ensemble models following the same modeling workflow, differences in prediction accuracy are attributed primarily to the number of occurrence records. The updated ensemble, built using a broader covariate set (*n* = 122), yielded improved predictive performance. These findings suggest that even with fewer occurrence records, robust species distribution models can be achieved through careful management of multicollinearity, integration of diverse algorithms, and ensemble modeling approaches.

## 4. Discussion

The global spread of *Aulacaspis yasumatsui* is largely driven by the international trade of Cycadaceae and Zamiaceae, and it has raised major concerns for both native and cultivated cycads. As such, the global expansion of *A. yasumatsui* is yet another expression of a relational geography where circuits of capital [65,66] shape a space for the invasion of species into new habitats, which can be harmful to native flora and fauna, in a pattern that is similar to what has been observed with pandemics and other emerging diseases [67,68,69]. Biologically, this spread occurs because the primary hosts are cycads (Cycadaceae, Zamiaceae), present as both native stands and widely planted ornamentals; urban gardens, nurseries, and wholesale hubs concentrate hosts and act as stepping stones that increase propagule pressure, shorten dispersal distances, and enable rapid establishment along trade and landscaping corridors.

Previous species distribution models (SDMs), primarily using the MaxEnt algorithm, failed to predict high suitability in regions such as Mesoamerica and parts of Africa [34,35], where the species is now established [9,22]. Our updated ensemble model, informed by a broader and more recent occurrence dataset and a refined selection of environmental predictors, successfully captures these newly invaded areas, particularly in Central America and the Caribbean, where suitable habitats in Mesoamerica and the Caribbean were predicted even with the reduced dataset of 86 records from a previous study [35].

A key factor driving the enhanced accuracy of our model is the integration of temperature-related covariates across multiple dimensions, reflecting *A. yasumatsui*’s complex climatic niche. Among these, the Mean Temperature of the Coldest Quarter (MTCQ) proved particularly critical, accounting for approximately 20% of covariate importance. The Logistic Binomial Model (LBM) revealed a sharp decrease in suitability above 3.5–4 °C, suggesting that populations are constrained by thermal thresholds during the coldest months. This sensitivity may explain the absence of established populations in colder temperate regions, despite apparent environmental suitability under less detailed models.

In contrast, the Temperature Annual Range (TAR) and Temperature Seasonality (TS)—both representing variability rather than absolute values—had subtler effects across models. However, both the LBM and Artificial Neural Networks (ANNs) showed increased occurrence probabilities at moderate TAR values (approximately 6–10 °C), which align with subtropical climates typical of Mesoamerica. These gradients suggest that moderate thermal oscillation, rather than stable tropical warmth or extreme fluctuations, may provide ideal conditions for establishment. Earlier models likely overlooked this nuance due to limited covariate resolution and algorithmic sensitivity.

The role of Actual Evapotranspiration (ACEV), which explained nearly 30% of the model’s predictive power, further supports this inference. High ACEV values, typically found in ever-wet equatorial forests such as the Amazon and Congo basins, were associated with reduced suitability. This helps explain why these regions remain largely free of *A. yasumatsui*, despite their apparent climatic favorability. Conversely, Climate Water Deficit (CLW) and Vapor Pressure (VAPRE), each contributing approximately 15%, showed positive associations with occurrence probability in several models, particularly the LBM and ANNs, suggesting that *A. yasumatsui* can persist under moderate water stress and high atmospheric aridity.

Taken together, the negative association with ACEV and the positive, saturating responses to CLW and VAPRE, captured most clearly by model algorithms sensitive to continuous gradients (ANNs, LBM), indicate that suitability for *A. yasumatsui* increases from per-humid toward sub-humid conditions and then plateaus near the dry margin.

The newly predicted suitability across Central America, including Panama, Mexico, and Costa Rica, reflects the convergence of these optimal conditions: moderate annual temperature ranges and intermediate water deficits. The previous failure to predict these regions can be attributed to both the narrower scope of environmental covariates and the use of a single algorithm (MaxEnt), which lacked sensitivity to complex interactions and nonlinear responses [38,39,42]. Within the sample sizes available here (tens to low hundreds), predictor choice and the multi-algorithm ensemble had a larger effect on performance than adding more occurrences. Even with the 86-record subset [35], models that explicitly represented cold-season temperature, moisture balance, and Actual Evapotranspiration and reduced predictors to a parsimonious seven recovered the Mesoamerican signal; by contrast, adding points in already well-sampled areas yielded diminishing returns after 5 km thinning.

For communication and planning, we translate continuous probabilities into three classes: high (>0.70), moderate (0.30–0.70), and low (≤0.30). We recommend (i) enhanced quarantine and nursery inspections in high-suitability areas, prioritizing ports of entry and ornamental-plant clusters; (ii) sentinel monitoring and community reporting in moderate areas to enable early detection; and (iii) baseline surveillance in low-suitability zones, with emphasis on pathways rather than blanket coverage. These classes can be mapped directly to guide phytosanitary surveillance and rapid response.

In contrast, our ensemble modeling framework, incorporating six different algorithms and weighing their outputs by performance (TSS > 0.5), offers a more robust and ecologically grounded representation of the Asian Cycad Scale niche. The improved model performance metrics (ROC = 0.95 ± 0.04; TSS = 0.86 ± 0.08) support the reliability of these predictions and highlight the value of multi-algorithm approaches for invasive species forecasting.

Future work aimed at better understanding the expanded geographic distribution of *Aulacaspis yasumatsui* might be improved by customizing the climatic data to the sampling period of species occurrence. To date, the vast majority of SDMs have been generated almost exclusively with the WordClim dataset [43], with little to no effort being made to include additional datasets with timeframes matching the timing of the occurrences. At the time of this paper being under review, the WorldClim dataset had been cited, according to Google Scholar, in over 16,000 studies despite large mismatches with the time of the modelled species occurrence data. This is conducted assuming climate stability, i.e., that estimates consider landscape elements that affect climatic dynamics [70], that estimates are based on long time series, and that proper resampling and robustness testing, e.g., varying geographical resolution and metrics for the evaluation model outputs, has been performed [71,72]. Thus, based on the current literature, we think our approach enriches common practice by using datasets whose uncertainties are properly understood and that also temporally overlap with the samples from the species whose distribution is being modelled. Ideally, data from remote sensing and estimates from climatic models that incorporate weather station data that coincide with the sampling of occurrences might improve the accuracy of the models. This could result in more precise estimates of habitat suitability based on present-day environmental data, and we are aware of at least one instance where such an approach has been pursued [57].

Furthermore, although our occurrence dataset was expanded and spatially thinned to reduce clustering, sampling bias and uneven geographic coverage still remain as potential concerns in our study, particularly in regions with limited survey efforts. This could have influenced model accuracy and ecological inference, as species distribution models (SDMs) are sensitive to biased presence data and rely heavily on the quality and representativeness of occurrence records [27,28,36]. Finally, the environmental covariates we employed captured key climatic factors; however, the models did not explicitly incorporate biotic interactions such as host plant availability, predation, competition, or mutualisms, all of which are known to strongly influence invasive species’ distributions [23]. The omission of these biological factors may limit the ecological realism and predictive power of the ensemble SDM, especially for a specialist herbivore like *Aulacaspis yasumatsui* that depends on cycads whose own distributions and vulnerabilities vary geographically.

## 5. Conclusions

Altogether, our findings emphasize the importance of temperature thresholds and variability, alongside moisture availability, in defining the potential range of *A. yasumatsui*. They underscore the need for dynamic SDMs that account not just for mean climatic conditions but for seasonal extremes and physiological tolerances, as organisms are sensitive to both mean environments and their variability [72,73]. These insights are crucial for risk assessment and the prioritization of conservation and biosecurity efforts, especially in biodiversity hotspots where endemic cycads are already under conservation threat.

## Figures and Tables

**Table 1:** Summary of the environmental covariates considered for the distribution modeling of the species *Aulacaspis yasumatsui* Takagi, 1977. Covariates are organized according to their importance (decreasing, presented in Figure 3B where abbreviations are matched with those presented under the “Abbreviation” column here).

Abbreviation	Covariate	Eco-Environmental Significance
ACEV	Actual Evapotranspiration (Derived)	Integrated energy–water flux; negative association; ever-wet per-humid climates correspond to lower suitability compared with sub-humid regimes
MTCQ	Mean Temp of Coldest Quarter	Winter constraint: suitability drops when MTCQ < ~3.5–4 °C, indicating cold-season limits on survival and establishment
CLW	Climate Water Deficit (Derived)	Unmet water demand; positive to a plateau; indicates tolerance of moderate moisture limitation, with saturation near the dry margin
VAPRE	Vapor Pressure	Atmospheric moisture (kPa); positive, saturating response that tracks the same moisture gradient as the deficit, peaking in sub-humid conditions and leveling toward the driest end
REV	Reference Evapotranspiration	Atmospheric evaporative demand (energy/temperature signal); helps distinguish sub-humid from per-humid settings
TS	Temperature Seasonality (sd × 100)	Captures thermal variability across the year; extreme seasonality reduces suitability
TAR	Temperature Annual Range	Annual amplitude of temperature; moderate ranges (~6–10 °C) align with higher suitability, whereas very low or very high ranges reduce establishment

**Table 2:** Comparison between the two models presented in Figure 3. The table shows receiver operating characteristic curve (ROC) and true skills statistic (TSS) values, with standard deviation (±SD).

Model	ROC	TSS
All (37 covariates)	0.90 ± 0.08	0.76 ± 0.13
Final (7 covariates)	0.95 ±0.04	0.86 ± 0.08

**Table 3:** Comparison of algorithm performance for model B using 112 observations vs. 86 observations from Wei et al. [35]. In the table: receiver operating characteristic curve (ROC), true skills statistic (TSS) values, and standard deviation (±SD).

Algorithm	Updated Model Evaluation		Wei et al. [35] Data Model Evaluation	
	ROC	TSS	ROC	TSS
GLM	0.97 ± 0.01	0.87 ± 0.01	0.83 ± 0.02	0.96 ± 0.05
RF	0.99 ± 0.13	0.91 ± 0.08	0.88 ± 0.03	0.97 ± 0.10
MARS	0.97 ± 0.03	0.83 ± 0.10	0.84 ± 0.01	0.98 ± 0.03
GBM	0.98 ±0.01	0.89 ±0.08	0.87 ± 0.02	0.97 ± 0.07
ANN	0.93 ± 0.05	0.82 ±0.12	0.80 ± 0.06	0.94 ± 0.15
CTA	0.91 ±0.04	0.81 ± 0.08	0.83 ± 0.05	0.92 ± 0.10

## Data Availability

The detailed location data are included in Supplementary Data.

## Supplementary Materials

Figures and raw data used in this article are available online at https://www.mdpi.com/article/10.3390/insects16101016/s1, Figure S1: Strip plot from previous studies; Table S1: Summary of environmental covariates; Table S2: Occurrence points.
