# Supplementary figures and images for: Ensemble Distribution Modeling of the Globally Invasive Asian Cycad Scale, Aulacaspis yasumatsui Takagi, 1977 (Hemiptera: Diaspididae)
Source: Insects. 2025 Sep 30;16(10):1016. doi: 10.3390/insects16101016 (PMC12564919; doi:10.3390/insects16101016)

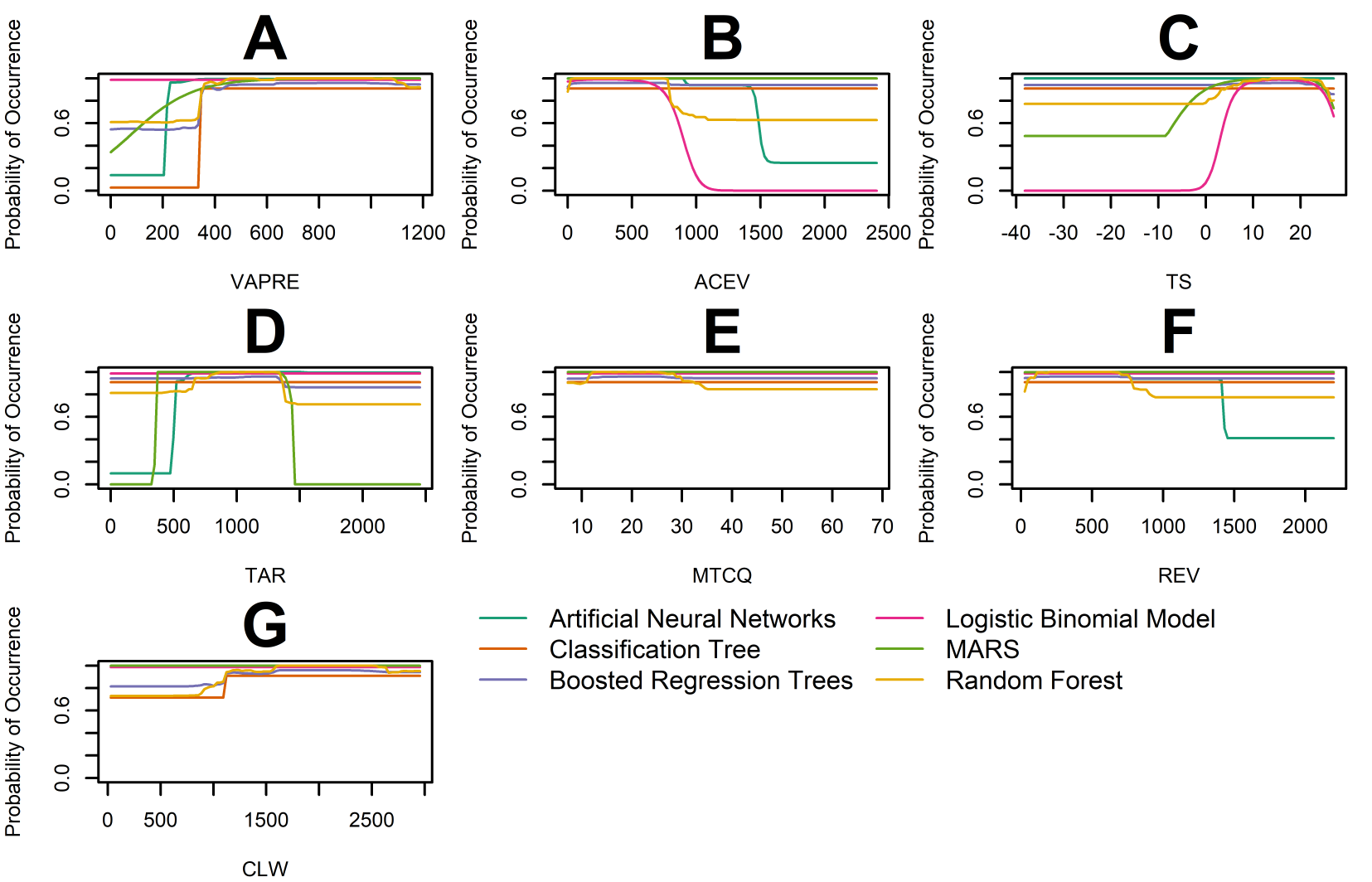

Supplement: Supplementary file 1 [file insects-16-01016-s001.zip › Figure S1 Strip plot from previous studies.tif]
